# Amide Activation in Ground and Excited States

**DOI:** 10.3390/molecules23112859

**Published:** 2018-11-02

**Authors:** Ervin Kovács, Balázs Rózsa, Attila Csomos, Imre G. Csizmadia, Zoltán Mucsi

**Affiliations:** 1 Institute of Materials and Environmental Chemistry, Research Centre for Natural Sciences, Hungarian Academy of Sciences, Budapest H-1117, Hungary; kovacs.ervin@ttk.mta.hu; 2 Laboratory of 3D Functional Imaging of Neuronal Networks and Dendritic Integration, Institute of Experimental Medicine, Hungarian Academy of Sciences, Budapest H-1083, Hungary; rozsabal@koki.hu; 3 Institute of Chemistry, Eötvös University, Budapest H-1117, Hungary; csomosattila@caesar.elte.hu; 4 Department of Chemistry, University of Toronto, Toronto, ON M5S 3H6, Canada; icsizmad@hotmail.com; 5 Department of Chemistry, Femtonics Inc., Budapest H-1094, Hungary

**Keywords:** amide, activation, amidicity, carbonylicity, transamidation, acyl transfer, excited state

## Abstract

Not all amide bonds are created equally. The purpose of the present paper is the reinterpretation of the amide group by means of two concepts: amidicity and carbonylicity. These concepts are meant to provide a new viewpoint in defining the stability and reactivity of amides. With the help of simple quantum-chemical calculations, practicing chemists can easily predict the outcome of a desired process. The main benefit of the concepts is their simplicity. They provide intuitive, but quasi-thermodynamic data, making them a practical rule of thumb for routine use. In the current paper we demonstrate the performance of our methods to describe the chemical character of an amide bond strength and the way of its activation methods. Examples include transamidation, acyl transfer and amide reductions. Also, the method is highly capable for simple interpretation of mechanisms for biological processes, such as protein splicing and drug mechanisms. Finally, we demonstrate how these methods can provide information about photo-activation of amides, through the examples of two caged neurotransmitter derivatives.

## 1. Introduction

The amide functional group is one of the most generally used moieties both in synthetic organic chemistry and bioorganic chemistry [1,2,3,4,5]. They have been known and studied for more than a century and can be commonly found in peptides/proteins and biologically active compounds, as well as in a broad range of synthetic drugs and toxins as a key functionality [1,6,7,8,9]. In general, when speaking of amides, the idea of a stable, unreactive group comes in our minds, however, they can play a significant role as reactants in a vast number of processes.

**Figure 1:**
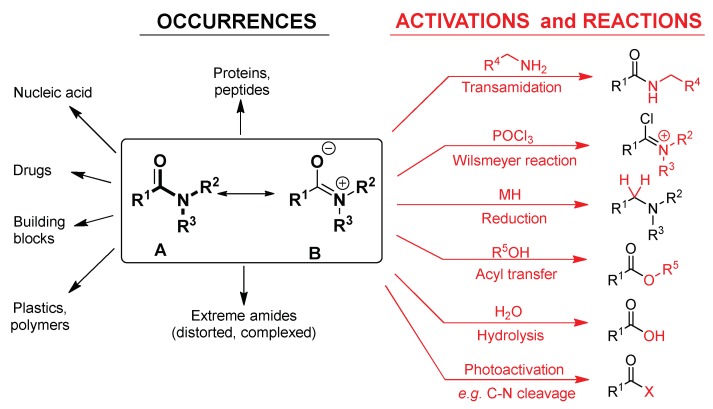
Contributing resonance structures of an amide bond (in the box). The occurrences (**left**) of the amide functionality and their typical reactions (**right**).

The wide variability in their reactivity can be attributed to the possibility of fine-tuning the bond strength. This can be controlled by attached substituent groups at the carbon and nitrogen atoms. The correlation between poor reactivity and planarity [10,11] of amides was proposed firstly by Pauling and later it was understood as being a result of the strength of the resonance-stabilized amide C–N bond [12]. Pauling predicted that a typical planar amide exhibits approximately 40% partial double-bond character, which renders the amide bond one of the most solid and least reactive functional groups (Figure 1) [13,14]. For example, the conjugation of an amide bond, which is traditionally specified as n(N) → π* (C=O), in formamide results in resonance energy (RE) of 91.29 kcal mol^−1^ [15,16]. In contrast to that, embedding the amide bond into a rigid bicyclic frame with nitrogen at the ring fusion, significantly modifies the degree of the resonance, therefore increasing the instability of the strained amide bond, for example in β-lactam antibiotics [17,18,19]. More intense conjugation implies a larger contribution of resonance stabilization, which increases the system stability thus lowering the reactivity and vice versa. In other words, the extent of conjugation predetermines chemical reactivity of the amides [20].

Amides can readily be cleaved by enzymes such as protease [15,16], but synthetically it is difficult to selectively break the carbon–nitrogen bond [21,22,23,24,25,26,27,28,29,30,31]. How can nature solve a problem easily, that is so hard for us? The answer is hidden in the process of activation of the unreactive group.

Organic chemists and “Nature” have developed methods to activate stable amide bonds, to make possible various reactions. Numerous examples of these can be found in the field of synthetic organic chemistry and biochemistry. The amide activation is typically based on the lowering of the amidic character by forcing the amide group to lose its conjugation. This can be achieved by substituent groups, as well as by forcing them into an inflexible structure. Moreover, as Thakkar et al. have described, the presence of the cis or trans isomer in the planar character of the amide bond in stable geometries can also influence the overall reactivity [32]. In connection with special chromophores, amide bonds can also be activated under excitation, as was demonstrated in some earlier studies [33,34,35]. Some of these processes include amidation, transamidation, reduction, coupling reactions by transition metallic complexes, and spontaneous or enzymatic hydrolysis.

The aim of our paper is to characterize the activity of amides using the amidicity and carbonylicity concepts and to demonstrate possibilities to increase or decrease their activity based on the bond strength [14]. In this work, using quantum chemical computation by using Gaussian 09 [36] at B3LYP/6-31G(d,p) level of theory [37], we reevaluated the data reported in the literature for reactivity of amide bonds. Here we reinterpreted some selected well-known compounds and reactions using the simple rule of amidicity, which can estimate the direction and rate of similar reactions involving more complex reactants.

## 2. Discussion

The amide group can be described generally by its resonance structures (A and B in Figure 1), where the nitrogen lone electron pair is delocalized between the nitrogen and the neighboring carbonyl. The condition of this phenomenon is a planar structure of the amide group. For the resonance structure B (Figure 1), the nucleophilic attack to the group is far less possible, thus making a general amide a resistant and unreactive group. The chemical and biochemical stability and reactivity is determined by the contribution of the minor resonance structure (B in Figure 1), in which formally a double bond is formed between the carbon and nitrogen atoms. These characteristics can be represented on a wide spectrum.

**Figure 2:**
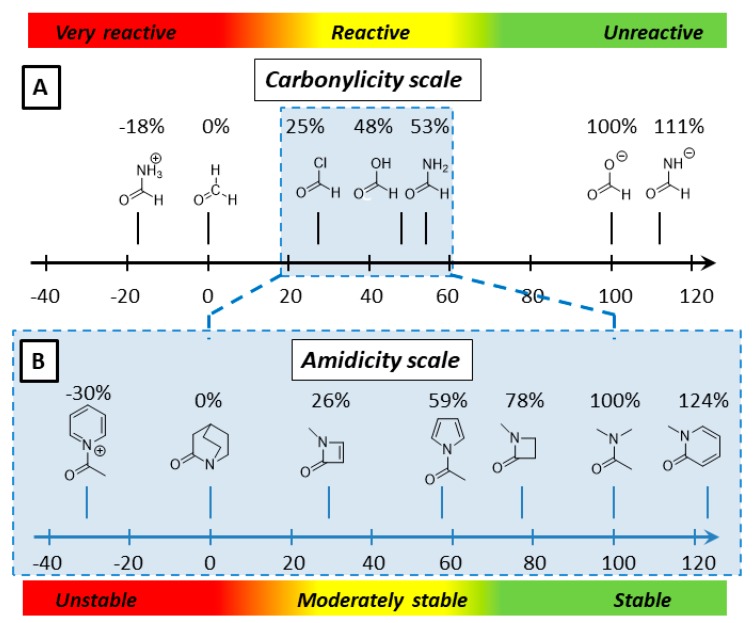
Typical carbonyl and amide compounds represented on the carbonylicity (**A**) and amidicity (**B**) scales. The direction of reactivity and stability increase are shown by red and green strips, respectively.

Earlier, the strength of a given amide bond was successfully characterized and the reactivity of different amides was made comparable to each other by creating the so called linear ”amidicity scale” (Figure 2B) [27]. Analogously, a linear “carbonylicity scale” (Figure 2A) was also constructed, extending the group of amides toward the entire world of carbonyl compounds, making all of them comparable [20]. Other methods, such as carbonyl substitution nitrogen atom replacement [6,38,39] and transamidation (TA) [14] which also aim to measure the amide stabilization energy, were also published in the literature. Using this scale, we are able not only to explain the reactivity of the amide bond, but also predict the effect of different substituents on the bond strength. Predicting amide bond strength effectively gives us the opportunity to activate or reactivate the desired amide bond. Manipulating the amidicity of a selected amide of a molecule has numerous synthetic applications. Also, calculating the amidicity—in other words, estimating the activity of the amide bond in biochemical compounds—may clarify the mechanism of action in which they are involved.

### 2.1. Amidicity Scale (AM%) and Carbonylicity Scale (CA%) and Their Resonance Enthalpies (H_RE_)

**Figure 3:**
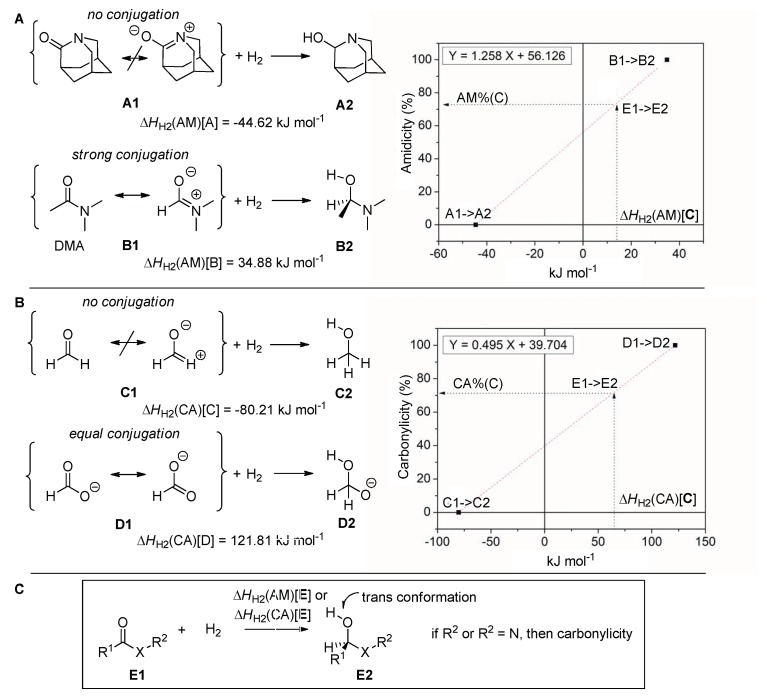
The definition of the amidicity (**A**) and carbonylicity (**B**) percentage based on the enthalpy of hydrogenation (Δ*H*_H2_) of the carbonyl group of a general carbonyl compound E1 (**C**). The two specifically chosen reference structures and their hydrogenation are also illustrated as A1 and B1 for amidicity (AM%; top) as well as C1 and D1 for carbonylicity (CA%; bottom). Values were obtained from the B3LYP/6-31G(d,p) geometry-optimized structures. In structure E2, the O–C–X–R_2_ and the H–O–C–X dihedral angles are chosen to be in the anti orientation.

The “amidicity scale” [27] quantifies the amide bond strength on a linear scale (Figure 3A), based on the computed enthalpy of hydrogenation (∆H_H2_) of the examined compound. A few examples positioned on the scale demonstrate the wide variety of amides. The scale is based on two specifically chosen reference compounds: A1 and B1 (Figure 3A). Compound aza-adamantane-2-on (A1 in Figure 3A) represents the lack of amidic charater, while dimethylacetamide (B1 in Figure 3A) corresponds to the perfect amide bond. In the structure of A1, the planar geometry is missing, due to the forced arrangement of the bridged N atom. To create the amidicity scale (AM%), the enthalpy of hydrogenation (∆H_H2_) of compounds A1 and B1 was calculated Equations (1) and (2) and amidicity was defined using a linear fit to these points. The enthalpy of hydrogenation for A1 (∆H_H2_[A]) is considered to have a 0% AM% value, while that for B1 (∆H_H2_[B]) is considered to have a 100% AM% value.

∆*H_H_*_2_[A] = *H*[A2] − {*H*[A1] + *H*[H_2_]} = −44.62 kJ mol^−1^ (1)

∆*H_H2_*[B] *= H*[B2] − {*H*[B1] *+ H*(H_2_)} *=* 34.88 kJ mol^−1^ (2)

As specified before, the carbonylicity scale (CA%) [20] quantifies the conjugation of the carbonyl group on a linear scale (Figure 3B), based on computed enthalpy of hydrogenation (∆H_H2_) of the examined carbonyl group (Equations (3) and (4)). Analogously to amidicity, the carbonylicity scale is also based on two specifically chosen reference compounds, C1 (formaldehyde; CA% = 0%) and D1 (formate; CA% = 100%) and defined using a linear fit to these points. Also, note that the amidicity scale is a defined section of the carbonylicity scale as it is represented on Figure 2, and differs only in the reference compunds chosen.

∆*H*_H2_[C] = *H*[C2] − {*H*[C1] + *H*(H_2_)} = −80.21 kJ mol^−1^ (3)

∆*H*_H2_[D] = *H*[D2] − {*H*[D1] + *H*(H_2_)} = 121.81 kJ mol^−1^ (4)

The linear fits are described by two pairs of fitting parameters, the slope (m_AM_ and m_CA_) and the intercept (b_AM_ and b_CA_). The values (AM% and CA%) for a desired amide or carbonyl bond can be easily calculated with the help of Equations (6) and (8), for a known enthalpy of hydrogenation, ∆H_H2_, from Equation (5). Both the amidicity and carbonylicity values can be transformed to the resonance enthalpy (H_RE_[E]; Equations (7) and (9)), which helps to estimate the energy benefit coming from the resonance inside the group. Neither of these scales is limited to values between 0% and 100%; in extreme cases the bond is weaker than A1 or C1 and stronger than B1 or D1, so they may display values outside of the aformentioned range. The amidicity scale can be considered as a sub-section of the carbonylicity scale.

∆*H*_H2_[E] = *H*[E2] − {*H*[E1] + *H*(H_2_)} (5)

*AM*%[E] = *m*_AM_ ∆*H*_H2_[E] + *b*_AM_ (6)

*H*_RE_[E] = *AM*%/*m*_AM_ (7)

*CA*%[E] = *m*_CA_ ∆*H*_H2_[E] + *b*_CA_ (8)

*H*_RE_[E] = *CA*%/*m*_CA_ (9)

### 2.2. Transamidation and Transacylation Reactions in Synthetic Organic Chemistry

**Figure 4:**
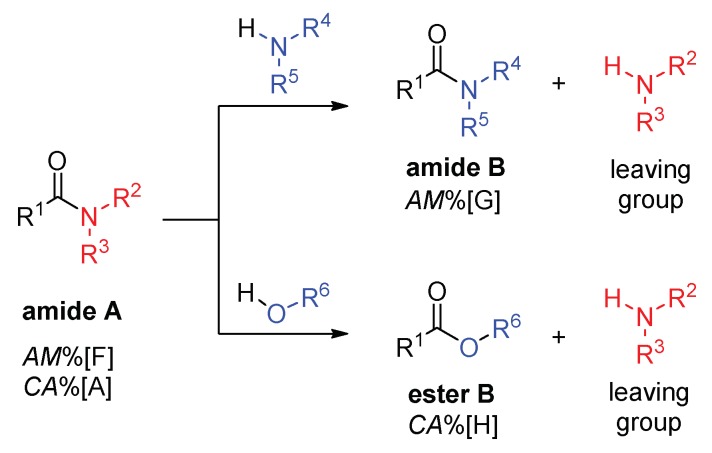
General transamidation reaction and the amidicity (AM%) and carbonylicity (CA%) percentages of the amides examined.

The amidicity value is useful to predict the outcome of a general transamidation reaction (Figure 4, top). The amidicity change ΔAM in Equation (10) or the change in stabilization enthalpy (ΔH_RE_ in Equation 12) leads to a thermodynamic selection rule, allowing for the reactions to be categorized as being either thermodynamically favorable or unfavorable. Generally, ΔH_RE_ is proportional with the entalpy change of the transamidation reaction. The lower the amidicity value of the chosen group, the greater reactivity it has towards nucleophylic reagents, like amines. The direction of the transamidation reactions can be predicted by comparing the sum of amidicities on the product and reactant sides. If the amidicity value increases, the reaction is energetically favoured in the given direction. If the difference is a negative value, the reaction is not driven forward. If there are different possible reaction routes, the one with the greater increase in amidicity is favored. The amidicity value is also capable of predicting the most active amide group in a molecule. Such a thermodynamic selection rule may also be used to predict the selectivity of a reaction in the presence of competing amide functionalities. This principle is illustrated as being operative in cases of different reactions. However, it should be mentioned that reactions may have several other parameters that determine if a reaction can proceed or not, such as steric hinderance, kinetic consequences, side reactions and the role of the leaving group on the acyl group. These amidicity and carbonylicity values describe only the thermodynamic background and not the kinetic aspect of a reaction.

Δ*AM = AM*%[G] − *AM*%[F] (10)

Δ*CA* = *CA*%[H] − *CA*%[F] (11)

Δ*H*_TA_ ≈ Δ*H*_RE_ = *H*_RE_[B] − *H*_RE_[A] (12)

**Figure 5:**
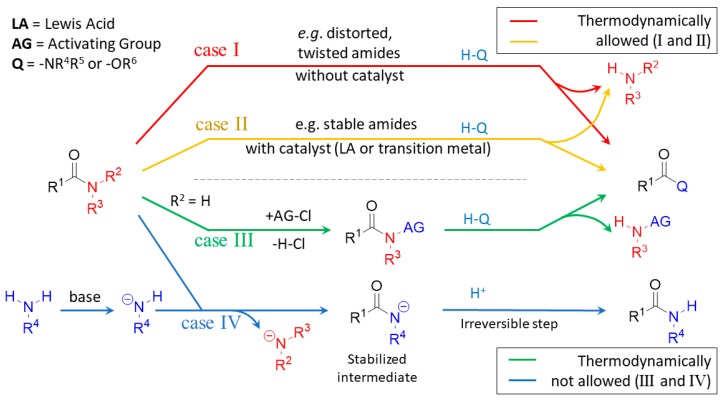
The classification of the acyl transfer and transamidation reactions into four cases (case I, II, III and IV), interpreted by amidicity (AM%) and carbonylicity (CA%) values.

On the basis of the exploratory screening of the literature, four cases were defined (Figure 5) [40]. Cases I and II (one-step processes) are both thermodynamically favored yet differ in reaction barrier height. Reaction barriers in case I are low [41], while those of case II are high, with the latter requiring a suitably chosen Lewis acid catalyst (AlCl_3_, BF_3_, TiCl_4_, HCl, etc.) to proceed normally [42,43]. This amine exchange reaction is sometimes carried out in a closed autoclave at high temperature by using the salt of the amine in the presence of BF_3_ [44], often to convert simple urea to a substituted one. In contrast to that, both cases of III and IV (two-step processes) are thermodynamically unfavorable, requiring the activation of either the reacting amide (case III) or the reacting amine. In case III, the amide is effectively destabilized, so activated by nitrosation, nitration, or acylation step [*N*-activation by activating group (AG), case III, Figure 5]. This activation is manifested as an increase in the energy level of the starting state, wherein the activated amide intermediate provides a thermodynamically favorable and rapid transamidation process. 

In case IV (*N*-deprotonation), a theoretically thermodynamically unfavorable transamidation, which may proceed in the presence of a strong base (NaH or NaOR). The product monosubstituted amide forms and stays in its deprotonated form (–CO–NHR^4^) and the starting amide is disubstituted non-deprotanable (–CO–NR^1^R^2^, amide). Therein, the forming amide-anion intermediate is stabilized by the deprotonation. In this case, an originally thermodynamically unfavorable transamidation process involves a thermodynamically favorable reversible subprocess, which finally finishes irreversibly by protonation in the acidic work-up.

#### 2.2.1. Examples of the Thermodynamically Allowed Transamidation Reactions (case I and II)

##### Transamidation Processes of Simple Amide Compounds

**Figure 6:**
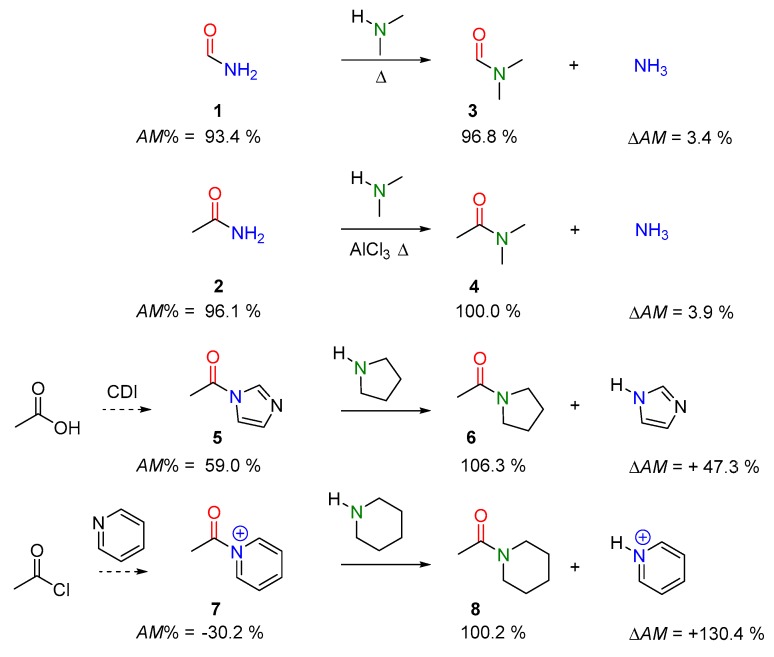
Examples for transamidation involving secondary amine, with the corresponding amidicity values computed at B3LYP/6-31G(d,p) level of theory.

In order to illustrate the essence of the transamidation reaction, we are demonstrating this concept by simple amides and amines. Unsubstituted amides, such as **1** and **2** in Figure 6, exhibit a moderately reduced value of amidicity (Figure 6) relative to mono-substituted or di-substituted amides, such as **3** and **4** (97–103%; Figure 6) [40], so one may therefore predict a transamidation process between them. Mono- and di-substituted amines (e.g., dimethylamine) are shown to react readily with formamide (**1**) at RT (25 °C) or above without any additional activation (case I). However, the transamidation reaction of acetamide (**2**) required elevated temperature and the presence of AlCl_3_ as a Lewis acid activator in order to attain an acceptable reaction rate (case II). The harsh conditions are explained by the high activation energy of the sterically hindered reaction center [40].

Compound **5** represents mild acylating agents (Figure 6) taking part readily in transamidation reactions with amines (e.g., pyrrolidine), forming amide **6** beside imidazole as a side-product. The synthesis of **5** can be carried out simply by means of the corresponding acid and carbonyl diimidazole (CDI) reagent. The increased acylating potency (AM% = 59%) of **5** can be attributed to the competition for the N atom lone pair between the imidazole aromatic ring and the carbonyl group of the amide. This phenomenon decreases the amidicity percentage of **5** to a value as low as 59% compared to normal amides. During the acylation reaction **5** → **6**, the amidicity value increased significantly, providing the main driving force for this transamidation reaction. Compound **7** (Figure 6) exhibits an extremely low amidicity percentage (−30.2%), making this molecule an excellent acylating agent, which can be prepared in situ from AcCl and pyridine. Consequently, **7** readily furnishes the acylation with an amine (e.g., piperidine), resulting in product **8**. Here, the reaction is motivated by the extremely large change in amidicity (∆AM, Figure 6) even at low temperature [24,31,40,45].

Amidicity value is also able to explain the inactivity and resistance of some commonly used organic amide solvents, For example, *N*,*N*-dimethylformamide (DMF, AM% = 100%) and *N*-methylpyrrolidinone (NMP, AM% = 136%) exhibit extremely high amidicity values. 

When dissolving any amine in these species, no reaction occurs between them due to the negative ΔAM, which makes them unfavorable for taking part in any reaction even at high temperature [24,31,40,45].

##### Transamidation and Acyl Transfer Processes of *N*-Phenylamides. 

In the literature, there are numerous examples where acylated *N*-alkyl anilines are reacted by amines or alcohols in the presence of transition metal catalysts (Pd, Ni, etc.). In these cases, the *N*-alky-anilids exhibit moderately low amidicity values, due to the dual effect of the phenyl ring. The phenyl ring suffers some steric hindrance and competes with the non-bonding electron-pair of the N atom against the acyl group at ones. In retrospect, it appears that the transamidation and acyl transfer reaction can occur without any catalyst, although, in the literature at different times, both of these types of reactions were reported as new approaches. Initially, these were reported to proceed without catalyst in the 1960’s [46,47,48,49]. However, analogue reactions were highlighted later as a novel approach in the presence of Ni or Pd catalysts. Authors called these reactions new, cross-coupling reactions of *N*-Ar amides (anilides), as reported by Garg et al. [50]. The reactivity of theses aromatic amides, under Ni-catalyzed esterification (Ni/SIPr) was rationalized by the concept of so called “barrier-free” rotation around the N–CO bond [51]. Analogues transacylation reaction with alcohol in the presence of Pd catalyst was also reported [52]. However, the earlier metal or catalyst-free transamidation solutions have subsequently been reinvestigated and rereported as new results [53]. This apparent confusion can be easily resolved by comparing the difference of amidicity values between the aromatic amides (anilids) with aliphatic amides, which thermodynamically allows such transamidation or acyl transfer reaction. 

**Figure 7:**
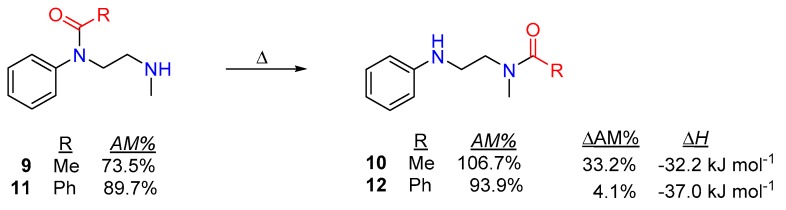
Typical example of intramolecular transamidation rearrangement, with the corresponding amidicity values (AM%) as well as amidicity (ΔAM) and enthalpy change (ΔH), computed at B3LYP/6-31G(d,p) level of theory.

A simple, but illustrative example can be found in the literature, which describes an intramolecular transamidation, where two amine-anilides (**9**,**11**) rearrange to aniline-alkyl-amide products (**10**,**12**; in Figure 7) without any catalyst. The positive changes in amidicity values as well as the exothermic enthalpy changes undoubtedly prove the efficiency of these reactions [46,54,55]. Other creative synthetic applications, taking advantage of the *N*-phenyl-acyl activation, were also developed for the synthesis of large lactams [53].

##### Acyl Transfer Reactions of Distorted or Twisted Amides

**Figure 8:**
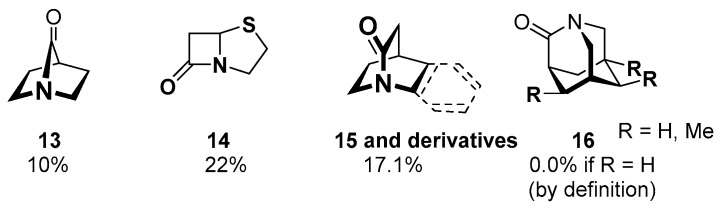
Typical examples of synthetized twisted amides (**13**–**16**) with the corresponding amidicity computed at B3LYP/6-31G(d,p) level of theory.

The structural distortion of an amide bond significantly modulates the degree of the amidic resonance, typically by lowering its amidicity/carbonylicity value [56,57]. The two classic twisted bridged lactams (aza-bicycloheptanone **13** [17], penicillin scaffold-penam **14** [58,59,60]) were synthetized more than 75 years ago. More than six decades later, this was followed by the preparation of **15** (in 1998) and its aromatic derivatives [61,62,63,64]. The aza-adamantane analogue **16** (in 2006 [65]), as well its substituted derivatives [66,67,68] made the series complete. These compounds furnish fully perpendicular arrangement within the amide bonds, resulting in high reactivity similar to that of ketones (Figure 8).

**Figure 9:**
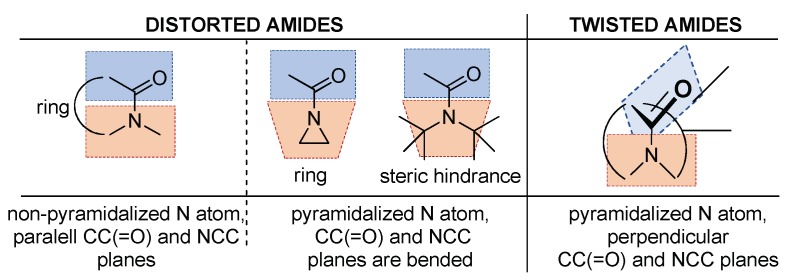
Redefined difference between the distorted and twisted amides. Distorted amides suffer steric hindrance or ring stress with pyramidalized or non-pyramidalized N atom. Twisted amides do not exhibit any strain, with perpendicular, non-overlapping amide planes. The carbonyl and amide planes are illustrated by the parallel- or perpendicularly-oriented blue and orange colors.

At first, we should clarify the difference between the distorted and twisted amides (Figure 9). Updating a previous nomenclature [6,69], the distorted amides reflect some steric hindrance or ring strain in their entire structures, but they already possess significant conjugation, with mentionable amidicity values. Here the amides are almost planar. In contrast, twisted amides may be described by a near perpendicular amine plane to the carbonyl plane with pyramidalized N atom, where the whole structure itself is not distorted or hindered (Figure 9).

Recently, twisted and distorted amide bonds have slowly found real application as a potent reagent in the mainstream of general organic chemistry. This reagent advances the highly reactive N–C amide bond in metal-free as well as transition metal catalyzed cross-coupling reactions yielding various carbonyl derivatives.

**Figure 10:**
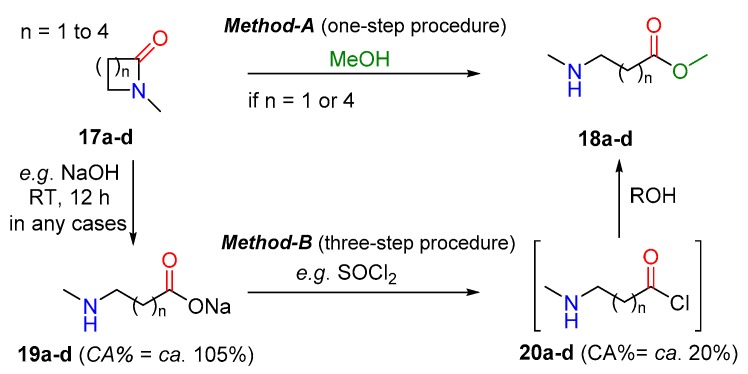
One step (Method-A; **top**) and multi-step (Method-B, **bottom**) procedures to transform cyclic amide (lactam) with various ring sizes to amino ester (for the corresponding carbonylicity values computed at B3LYP/6-31G(d,p) level of theory; see Table 1). The carbonylicity values for 19 and 20 are average values.

Distorted ring-amide can be represented generally by the correlation between the cleavage of lactam rings and the ring size. In Figure 10 and the corresponding Table 1 we summarize the possible alcoholysis of four to seven-membered lactams (n = 1–4) to open chain amino ester in the light of the calculated carbonylicity values. It is important to clarify, that the CA% values are corrected with ring strain as described. The smallest ring-sized lactam (**17a**; n = 1 in Figure 10) has relatively low carbonylicity value (40.5%), which may be due to the insignificant conjugation and not only to the ring strain (Table 1). Thus, **17a** can transform to the corresponding open-chain amino ester **18a**, already having a normal value (ca. 56%; Figure 10, Method-A). Generally, five- and six-membered rings (**17b**, n = 2 and **17c**, n = 3) are the most stable among the small ring lactams, so one-step alcoholysis reactions are not allowed. More precisely, for five-member ring lactam **17b** this one-step process toward **18b** is forbidden due to the negative change of the carbonylicity value (ΔCA = −3%), while in the case of six-member lactam the ring opening process (**17c** → **18c**) exhibits an almost neutral change (ΔCA = +1%). In these cases, a more complicated multi-step processes (**17** → **19** → **20** → **18**; Method-B) should be used to cleave the lactam ring effectively. In this longer process, the hydrolysis with NaOH is allowed due to the increasing CA% (**19b**,**c**). The acid chloride formation (**20b**,**c**) activates their functionalities, which easily transform to the desired ester **18b**,**c**. Finally, the seven membered lactam (17d; n = 4) has a slightly lower carbonylicity value (53%), which allows a moderate potency to transform it to **18b** in alcohol in one step, with slightly increasing ΔCA. It should be mentioned that the longer procedure **17** → **19** → **20** → **18** is available for all ring sizes. 

**Figure 11:**
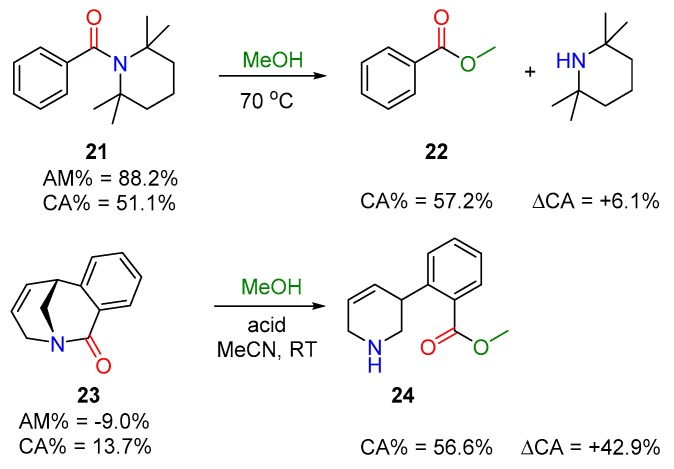
Relevant examples for acyl transfer reactions in the cases of distorted (**21**) and twisted amides (**23**) resulting esters (**22** and **24**), explained by the change in carbonylicity (CA%) with the corresponding carbonylicity and amidicity values computed at B3LYP/6-31G(d,p) level of theory.

Some examples of sterically hindered amides were reported [70,71,72] to exhibit moderately low amidicity and carbonylicity values. One representative example is **21**, where the amide functionality is sterically hindered and, hence, distorted. Here, somewhat decreased carbonylicity makes the reaction possible with alcohol (for example MeOH) to yield an ester (**22**), as published earlier (Figure 11, top) [73]. Another example shows that a twisted amide (e.g., **23**), having an extremely low amidicity value, readily cleaves its CO–N bond by MeOH, resulting an open-chain amino-ester (**24**), as shown in Figure 11 (bottom). In both cases, the thermodynamic driving force can be easily explained by the positive change in carbonylicity [74,75]. The combination of the twisted amide and acyl-anilide concepts are also exemplified, where these amides furnish effective alcoholysis analogously to the previous cases [73,76].

#### 2.2.2. Transamidation Reaction via Activated Amides (case III)

**Figure 12:**
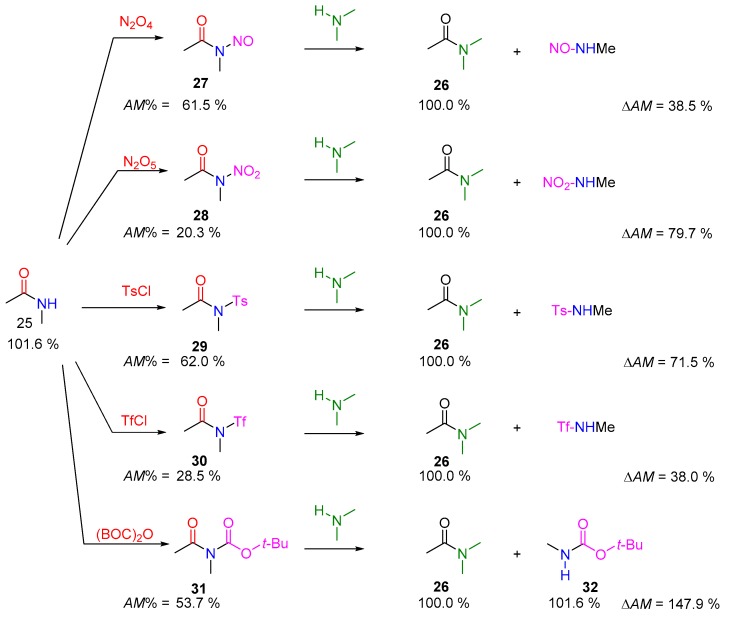
Some representative examples of amide activations (case III), using various reagents with the corresponding amidicity values computed at B3LYP/6-31G(d,p) level of theory. Ts = *p*-toluenesulfonyl; Tf = CF_3_SO_2_–; BOC = *t*–BuO–CO–.

Non-strained secondary amides (e.g., *N*-metylacetamide; **25**) exhibit approximately 100% amidicity, so are typically not able to take part in transamidation reactions. For instance, the reaction between **25** and dimethylamine, yielding theoretically dimethyl acetamide (**26**), is not beneficial, due to the slightly smaller amidicity [40]. From the perspective of practicing chemists, there was a synthetic demand to activate and use these amides as reagents. To activate these resistant secondary amides, their high amidicities should be decreased by an activating group. Numerous methods were developed in recent decades and presented in the literature, summarized in Figure 12. All these solutions involve a preliminary activation step. 

The introduction of *N*-nitroso (**27**) [77,78], *N*-nitro (**28**) [79], *N*-(tosyl) (**29**) [80], *N*-(trifluoromethanesulfonyl) (30) [80], or *N*-acyl (31) [81,82,83] amides are the most relevant examples. The following step describes how these activated amides (27–31) could react with, e.g., dimethylamine, to yield the desired and exemplified amide product 26. The byproducts of these reactions are typically good leaving groups. The most significant amidicity decrease can be measured for **28** and **30**, producing the most reactive acylating agents [79,80]. The *N*-BOC-*N*-methyl-acetamide **31** was prepared in a previous step from **25** by (BOC)_2_O reagent [82,83], a process known as *N*-Boc activation. Other, bisacyl and triacyl substituted amides (such as succinimide [84] and phthalimide derivatives [85]) may also behave as good transamidating agents in the presence of an amine. In all cases, the listed ΔAM values were large enough to provide enough driving force to convert to the desired product **26** [40].

**Figure 13:**
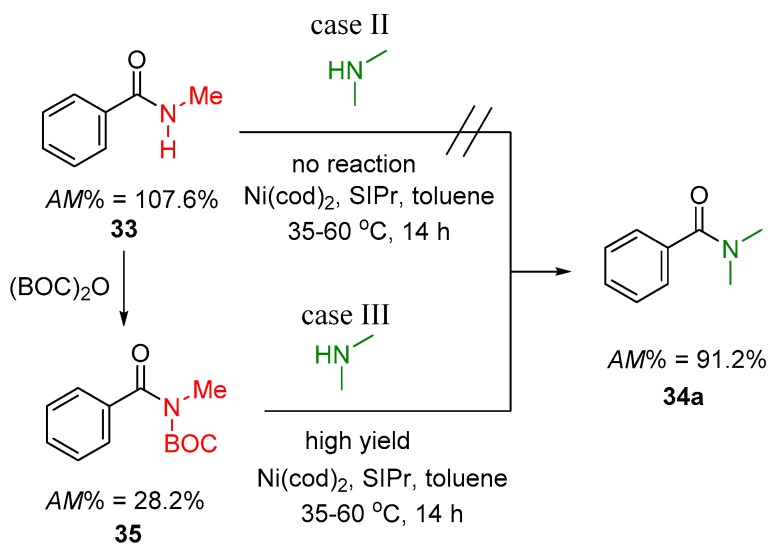
Exemplified transamidation reaction of **33** (case III) with (**33** → **35**) and without amide activation. The corresponding amidicity values were computed at B3LYP/6-31G(d,p) level of theory. BOC = *t*–BuO–CO–; cod = 1,5-cyclooctadiene; SIPr = 1,3-bis(2,6-diisopropylphenyl)imidazole-2-ylide.

Finally, a good example can be found for the transition metal catalyzed transamidation, where *N*-alkyl-benzamide (e.g., **33**) was transformed to dialkyl derivatives (e.g., **34a**). The authors previously reported that **33** could not be transformed to **34a** directly, even in the presence of an active transition metal catalyst (Ni/SIPr), without providing any theoretical interpretation. However, the amidicity concept is able to highlight the thermodynamic background of the process failure, due to the unfavorable amidicity change (Δ*AM* = −16.4% in Figure 13). Nevertheless, with the activation of the amide by BOC group (**35**), the progress occurs smoothly to product **34a**, in the presence of Ni/SIPr catalyst. It seems reasonable to conclude that when the transamidation reaction exhibits positive amidicity change (as in **35** → **34**), the transition metal catalyst or any other catalyst may help. However, when the reaction is substantially endothermic (as in **33** → **34a**), the reaction will not take place even if catalyst is used.

**Figure 14:**
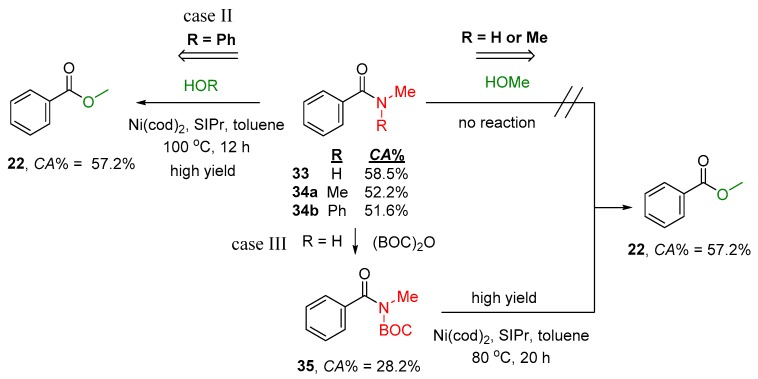
Exemplified acyl transfer reaction (case II and case III) with and without amide activation in the presence of catalyst. The corresponding carbonylicity values were computed at B3LYP/6-31G(d,p) level of theory. BOC = *t*–BuO–CO–; cod = 1,5-cyclooctadiene; SIPr = 1,3-bis(2,6-diisopropylphenyl)imidazole-2-ylide.

Analogues reactions can be found in the literature, where variously substituted amide compounds are used as a reagent to convert them to ester derivatives with the corresponding alcohol. Some examples are summarized in Figure 14, where substituted benzamide derivatives (**33**–**34a,b**) could be converted to **22**. Clearly, these reactions can occur, if the carbonylicity difference is favorable. In the case of the transformation of **33** (R = H) to **22**, the reaction is forbidden due to the negative change in carbonylicity, with or without catalyst (Figure 14). The transformation of **34a** to **22** is theoretically possible, but the reaction condition applied in the publication was not harsh enough to be effective (Figure 14, case II). In the case of Ph substituents (**35**), however, maintaining low amidicity value is sufficient to enable the acyl transfer in the presence of Ni/SIPr catalyst [50]. Finally, **33** can transform to **22** effectively by the activation method with (BOC)_2_O reagent (Figure 14, case III). The prepared active intermediate **34b**, having a low amidicity value, finally can be transformed to the ester product, in the presence of Ni/SIPr catalyst.

From these examples it becomes clear that there is no fundamental difference between transamidation reaction and other transacylation, because in both cases there is an acyl migration. However, for historical reasons, the transamidation and acyl transfer processes are still distinguished in the literature. Needless to say, all of these conclusions are true when the heteroatom is sulfur. 

The reverse pathway with different substituent patterns, the transacylation from ester to amide, was also described in the literature in the presence of Ni/SIPr catalyst [86,87,88].

#### 2.2.3. Activated Transamidation Reaction via Product Stabilization [case IV]

**Figure 15:**
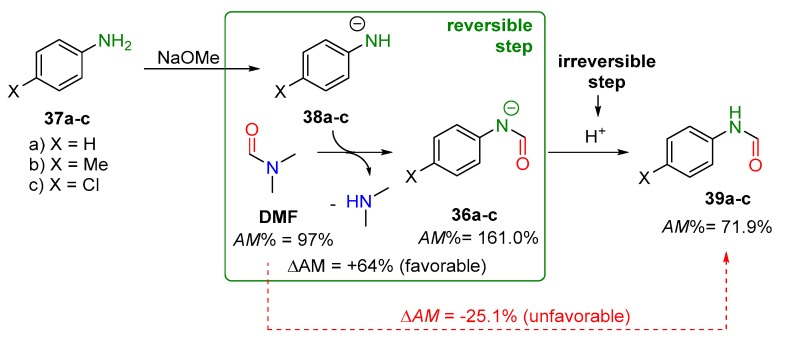
An example for case IV transamidation reaction with the corresponding amidicity values computed at B3LYP/6-31G(d,p) level of theory. The green box illustrates the thermodynamically favorable subprocess, while the red arrow shows the overall unfavorable reaction.

Because of its high amidicity value, *N*,*N*-dimethyl formamide (DMF; Figure 15) can be considered an unreactive amide. In contrast to this, deprotonated amides, such as **36**, possess even higher amidicity values (>150%) than tertiary amides. In strongly basic conditions, deprotonated amides (**36**) should, therefore, be more favorable than non-deprotonable DMF. A good example is found in the literature, where aniline derivatives **37a**–**c** can be deprotonated by NaH or NaOMe, generating their corresponding anion form of **38a**–**c**. The product amide anions **36a**–**c** were formed at elevated temperature (Figure 15) [36,89,90,91]. During the workup process, an aqueous acid was used to acidify and precipitate the neutral product **39a**–**c** in an irreversible way. The procedure as a whole is a thermodynamically forbidden transformation (**38** + DMF → **39**), due to the lower amidicity of the product **39a**–**c**. Nevertheless, only the reversible subprocess of **38** + DMF → **36** (in green box, Figure 15) is favorable. Overall, the driving force of this subprocess is controlled by the high ΔAM, so this formylation reaction is rapid, making this reaction practical [40].

**Figure 16:**
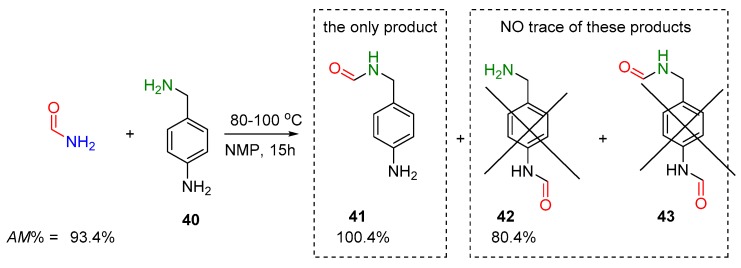
Selectivity of transamidation reaction of *N,N*-dimethylformamide (DMF) and compound **40**, having two types of amino groups. The three possible products **41**–**43** are shown. The corresponding amidicity values were computed at B3LYP/6-31G(d,p) level of theory.

The use of the amidicity change (ΔAM) leads to a quasi-thermodynamic selection rule, allowing the reactions to be categorized as being either thermodynamically favorable or unfavorable. This simple rule of amidicity-change may be used to predict the selectivity of a reaction, where two competing functional groups are involved. Such an example is presented, where two types of amino groups are attached in one molecule (**40**). As one may predict by the amidicity change, only the alkyl amine group will react with formamide, yielding **41** exclusively with high yields in one step (Figure 16). The other isomer amide **42** and the diacylated **43**, including anilid type amide, could not be detected in the reaction mixture, as the amidicity change simply predicted [40].

### 2.3. Amide Reduction via Amide Activation

An unprecedented number of examples can be found in the literature to reduce amides to substituted amines, using numerous reduction agents (most generally metal hydrides). The situation is illustrated by a few selected citations [31,69,92,93,94,95,96,97]. Strong reduction agents are a common feature of these methods, since generally amides are very resistant against reduction. Consequently, these methods are typically not applicable to carry out selective reactions. This extreme resistance can be explained by the strong conjugative interaction within the structure of amide, exhibited by their high amidicity values. Among the reduction agent (LiAlH_4_ or BH_3_), the complex and highly active metal hydrides may have the most significant synthetic importance to obtain various amines.

**Figure 17:**
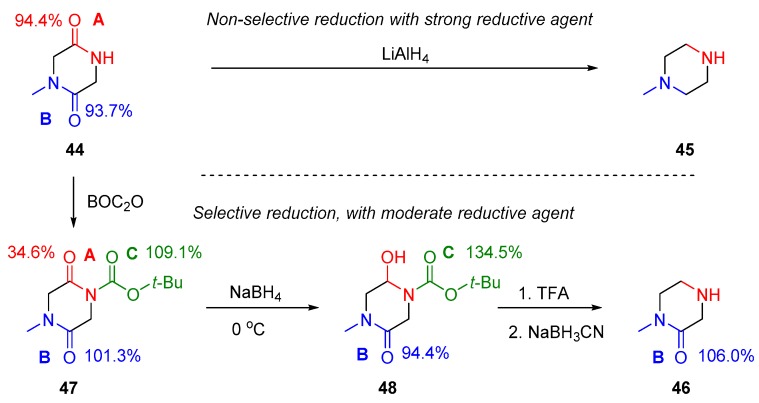
The non-selective and selective reduction of the amide bonds in a diketopiperazine derivative **44**, including amide-A and B, to yield **45** and **46**. The corresponding amidicity values were computed at B3LYP/6-31G(d,p) level of theory.

However, selective examples can be found in the literature in which one of the amide groups was reduced selectively in one molecule, while keeping the other amide or other sensitive groups of the molecule untouched [98,99,100,101]. This was achieved by the activation of the selected amide group by an appropriate *N*-substituting activating group. The following example illustrates this methodology in Figure 17, among other examples. The case of the exemplified diketopiperazine derivative **44** [101] includes two unequal but very similar amides, A and B, from the aspect of amidicity. If one tries to reduce this compound **44** by LiAlH_4_, both the amides A and B will be reduced to piperzine derivative **45**. From this reaction it would become clear for a practicing chemist, that no possibility exists of finding a selective reducing agent to obtain piperazidone derivative **46**. In the case of adding only one equivalent of reductive agent, a complex mixture of partially reduced forms can be observed.

However, analogously to case III reactions in the previous section, the secondary amide-A was acylated by BOC_2_O reagent, which resulted in a triacylated intermediate **47**. In this structure, the amidicity of the acylated amide A is lowered to 34.6%, while that of amide B increased slightly. The carbonyl of the BOC group (amide-C) is as high as 109.1%, which exhibits large resistance to reduction. The lowered amidicity of the amide-A provides not only higher reactivity with the metal hydride, but allows the application of significantly weaker reducing agent, such as NaBH_4_. This reagent is soft enough to avoid the reduction of amide-B and C. In the first reduction step, amide-A is reduced only to hydroxy amine intermediate **48**, protected by the BOC group, but with increased amidicity for amide-C. This intermediate **48** was transformed finally to the desired product **46** by a consecutive deprotection and reducing steps. This compound later proved to be an excellent building block in drug research. This method is not limited only to six-member diketopiperazines, but it can also be generalized for even open chain systems [101].

### 2.4. Amide Reaction in the Biochemistry

Natural products are very precisely assembled from selected molecular components in unique arrangements. They typically exhibit very effective and targeted mechanisms of actions in numerous biological processes. There is an observably large difference in complexity, activity and efficiency of human-designed compounds [102] and nature’s biomolecules, such as dinucleotide coenzymes (NAD and FAD) [103], calicheamicin-γl [102,104,105], duocarmycin [106,107,108], syringolin A [18], aflatoxin B [18], and penicillin [109]. Biochemical processes also take advantage of the selectivity of acyl transfer reaction and amide activation, so three representative examples are presented in this section.

#### 2.4.1. Cross-Linking in the Blood Clotting Process

**Figure 18:**
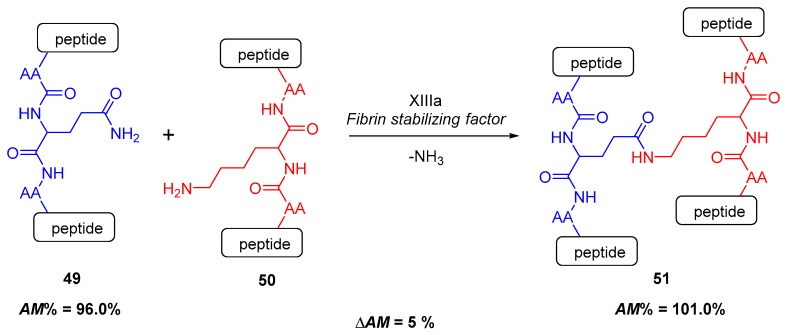
The final step of the blood clotting process from the aspect of amidicity change, catalyzed by the transamidinaze enzyme. The corresponding amidicity values were computed at B3LYP/6-31G(d,p) level of theory.

The first example is taken from the multistep process of blood clotting [110,111,112]. In this case, in the final, thirteenth step (Figure 18) of the entire complex process, the two final protein intermediates **49** and **50** are linked to each other via a new side-chain amide bond [112]. 

This creates a molecular polymeric net (**51**), which forms a barrier against bleeding. Analyzing the process from the aspect of amidicity values (Figure 18), this transamidation reaction is spontaneous. The process takes part between a glutamine side chain amide (blue) and a lysin side chain amine (red). The amidicity change is predicted to be slightly positive (∆AM), because the initial 96.0% amidicity of the glutamine side chain amide is increased to 101% as the new amide is formed, not requiring additional activation. This small change in amidicity provides a driving force for this bioreaction, but it is not enough to exhibit the measurable high reaction rate at body temperature. Consequently this process is catalyzed by an enzyme fibrin stabilizing factor or transglutaminase [112,113]. Moreover, the releasing ammonia also makes the process irreversible.

#### 2.4.2. Intein-Mediated Protein Splicing 

**Figure 19:**
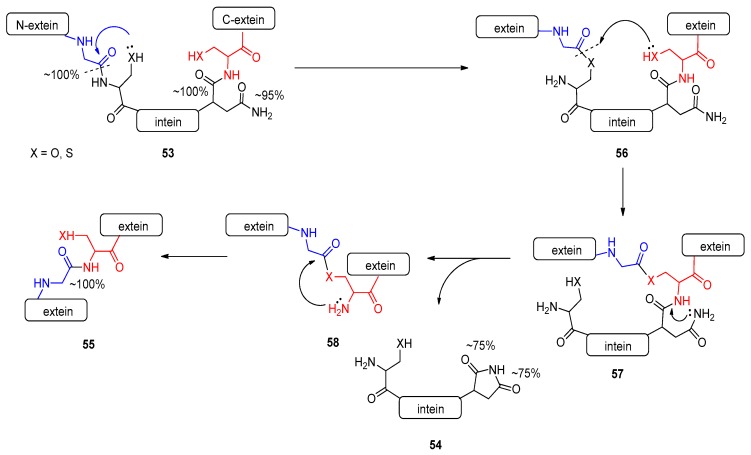
The mechanism of action of protein splicing as a special type of transamidation reaction.

Intein-mediated protein splicing is a biologically important process (Figure 19) [114,115,116,117], where a small but defined part of the protein **52**, intein (**53**), is cut out specifically from the middle of a protein. Meanwhile, the two remaining parts, called exteins, are linked to each other, forming a new protein **54** via a new peptide bond. Protein splicing is so rapid that the precursor protein is rarely observed in native systems. It was shown that the process is auto-catalytic and folding-dependent [118,119,120]. The N-extein residues play unique and important roles in protein splicing [120]. The intein peptide sequence is supposed to contain no sufficient information originating from an external source [114,115,116,117].

From the chemical aspect, here two amides are involved in transamidation process rather than an amide and an amine shown previously [33]. The intein and the C-terminal extein residue contain sufficient information for splicing in proteins, which involves four basic chemical steps. In the first step of the mechanism [114,115,116,117], the serine or cysteine residue, at the N-terminal border of the intein, attacks the neighboring peptide bond forming an intermediate **55**, which in a subsequent step rearranges to **56** and **57**. In the final step, the intein leaves the native sequence resulting in the edited protein **54**. Taking into account only the starting and ending state, the overall process exhibits a large negative ∆AM value (−45 %), which may suppose an endothermic reaction. One may argue that the exothermic refolding of the instantly cut intein sequence provides additional driving force, covering the energy demand of the overall amidicically unfavorable process. Moreover, the N-extein-intein amide linkage is a typically distorted amide (blue in Figure 19.) due to the folding, resulting in lower amidicity, which could initiate the acyl migration (blue arrow). 

#### 2.4.3. Penicillin

**Figure 20:**
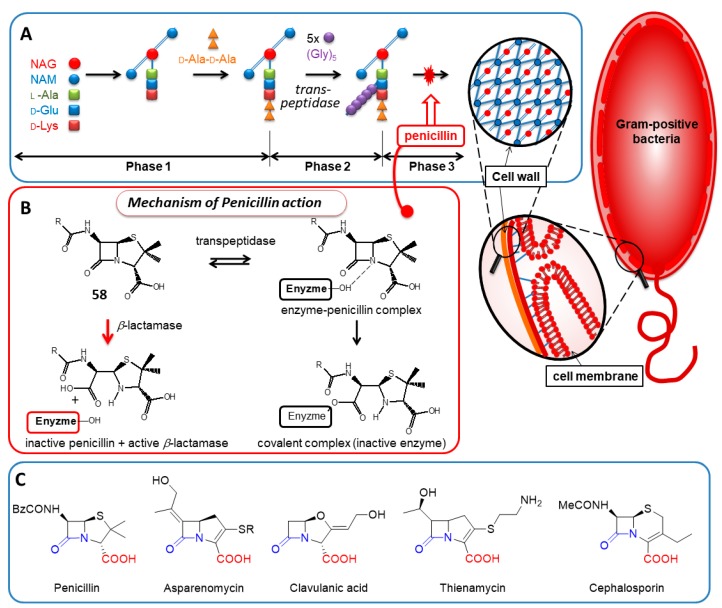
(**A**) The schematic mechanism of biological action of the penicillin antibiotic in the course of cell wall synthesis. (**B**) Penicillin inhibits transpeptidase enzyme by bonding covalently to the serine side-chain producing non-hydrolysable enzyme-substrate complex, blocking irreversibly the enzyme activity. (**C**) Some representative examples of β-lactam antibiotics. Their common feature is the strained β-lactam ring neighbouring a carboxylic functionate, which are both essential for the antibiotic activity. NAG = *N*-acetylglucoseamine; NAM = *N*-acetylmuramic acid.

One of the most important small biogen amides is penicillin (58), produced by the *Penicillium* genus, which is considered to be one of the great discoveries of the 20th century [121,122]. This antibiotic inhibits penicillin binding proteins, such as transpeptidase, by blocking their serine residue via acylation in gram-positive bacteria. In this way, bacterial cell wall synthesis stops, leading to deadly susceptibility to osmotic effects and cell bursting. This bacterial enzyme plays a crucial role in transferring the d-Ala-d-Ala dipeptide into the bacterial cell wall synthesis as illustrated by Figure 20. The perennial war between bacteria and fungus led to the evolution of β-lactam related antibiotics, and resulted in many variants, of which cephalosporine [123] and thienamycin [124,125] are just two examples.

During the last century, resistant bacteria strains have developed some defending mechanisms against intensively and overused β-lactam antibiotics. Consequently, antibiotic research is once again at the forefront of drug research. The β-lactamase enzyme is synthesized by resistant bacteria for deactivation of penicillin-type compounds in order to avoid their lethal effects [122,126,127,128,129]. Regardless, continued characterization of the β-lactam structure and mechanism aids in the understanding and design of novel antibiotics with desired effects and great precision [126].

Since the discovery of penicillin’s chemical structure [18,129], the way this molecule avoids the aqueous hydrolytic effects in body fluid, while immediately awakening its hidden ability to acylate the transpeptidase’s serine side-chain oxygen, has remained a mystery [130,131]. Recently, bacterial resistance has been investigated intensively, wherein penicillin hydrolysis was studied [131,132,133,134,135], and on the bases of this mechanism its activity was understood [132,133,134]. The “strange” fused β-lactam-type structure [132] was dedicated to explain its strength as an acylating agent completely, approximated earlier to that of acyl chlorides [133,134]. In organisms, however, such a strong acylating agent should decompose quickly before reaching its destination, e.g., the transpeptidase enzyme of the bacteria.

**Figure 21:**
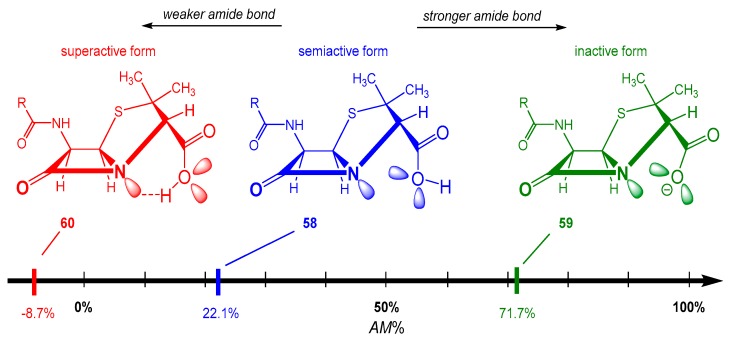
The amidicity scale [MP2(full)/DGDZVP], showing the reactivity of the carbonyl group for the three different forms of penicillin molecule (58, 59 and 60), depending on its protonation degree as well as its conformation. The electron repulsion between the COO− group and the N atom is illustrated by the lone pairs. This strengthening of the amide bond in 59 (larger amidicity) decreases reactivity with respect to 58. In form 60, the internal H-bond withdraws density from the amide bond, weakening it (extremely low amidicity) leading to extremely high reactivity toward nucleophiles.

Previously, it was shown that the unexpected stability of penicillin can be attributed to its deprotonated form (**59** in Figure 21), which is the dominant component of the neutral aqueous environment [60]. According to the new, higher level quantum chemical calculations at MP2(full)/DGDZVP, the amidicity value [40] of the anion form was found to be higher (AM% = 71.7%) than the neutral form (**58**), referring to the more stable amide bond in the anionic form. However, when the penicillin reached its target enzyme, the transpeptidase, it gets protonated (**59** in Figure 21) by the enzyme. The forming neutral form of penicillin (**58**) immediately lowers its amidicity to 22.1%, so it has been triggered for the reaction. Finally, the H atom on the carboxylic acid group can also easily turn back to the pyramidaziled amid N atom, forming an H-bond. This conformational change turns on the final, superactive form of the molecule (**60** in Figure 21), reflected by the extremely low amidicity (AM% = −8.7%). This form instantaneously makes a covalent enzyme-drug complex, and knocks out the enzymatic activity. This carboxylic function makes the molecule a well-designed acylating bacteria killer, because it also acts as a bait for the essential bacterial enzyme. In this way, penam scaffold antibiotics are practically self-activating selective hitmen against bacteria. In summary, penicillin provides an interesting example of a controlled protonation and conformation-dependent amide bond activation. 

### 2.5. Amide Activation in Excited State.

**Figure 22:**
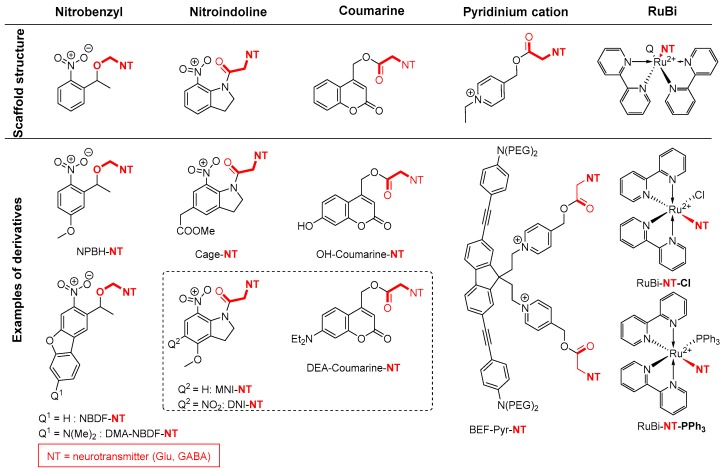
Collection of the most prominent cage-scaffolds (**top**) and some derivatives (**bottom**). NT = neurotransmitter. Compounds in the box are the subject of discussion.

The electronic excitation of the chromophore functionality is able to transfer the energy toward a neighboring amide bond, resulting in an amide activation and in an increased reactivity of the amide bond, which is utilized for various processes [33]. Photocleavage (also known as uncaging) is based on this phenomenon, providing a methodology to release selectively a bioactive compound from its covalently bonded cage-form in a localized volume. Otherwise, the cage scaffold should effectively block the activity of the bonded biomolecule, but undergoes efficient removal after excitation of the moiety, through the scissile bond. Until now, numerous cage molecules have been developed (Figure 22); however, nitro-indoline and coumarine scaffolds are the most popular of these, and are usually linked with glutamate as excitatory neurotransmitter [136,137].

#### 2.5.1. Photochemical Release of Glutamate from MNI-Glu and DNI-Glu at Excited State

**Figure 23:**
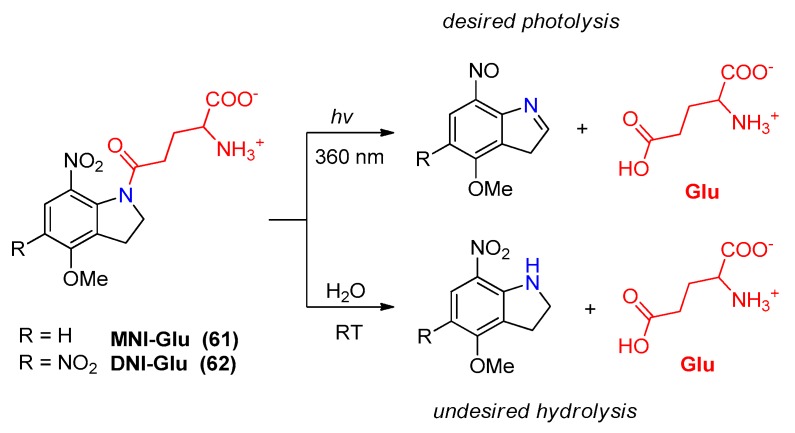
Chemical structures of MNI–Glu (R = H) and DNI-Glu (R = NO_2_) and their photochemical reactivity (**top**) and hydrolytic reactivity (**bottom**).

The most popular cage compounds, 5-mono-nitro-indoline (MNI) and 5,7-dinitro-indoline (DNI), have proved their effectiveness during the last decade and have been widely applied in numerous neuroscientific papers. Due to the large number of scientific results, in this paragraph we focus only on DNI-Glu and MNI-Glu [138]. MNI-Glu and DNI-Glu fulfill the main requirements for an ideal cage compound, including an efficient uncaging process with relatively high chemical yield, rapid two-photon induced release (quantum yield 8.5%) [139], low spontaneous hydrolysis rate, (Figure 23) and low biochemical side effects [140]. These properties were demonstrated to be useful in neurophysiological experiments.

**Figure 24:**
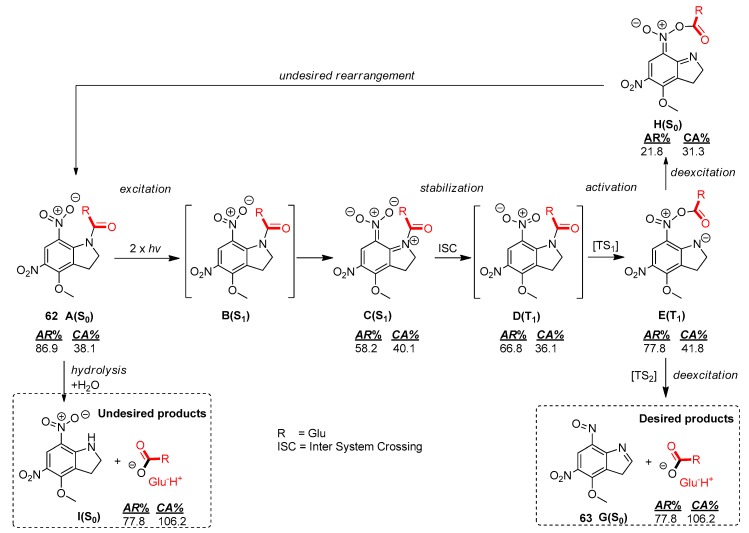
Quantum chemical modelling of the photochemical reactions as well as the simplified mechanisms of ground state hydrolysis for DNI-Glu (**62**). The aromaticity (AR%) of the benzene ring and carbonylicity of the carbonyl functionality along the mechanism are computed at B3LYP/6-31G(d,p)//PCM(water) level of theory [37].

**Figure 25:**
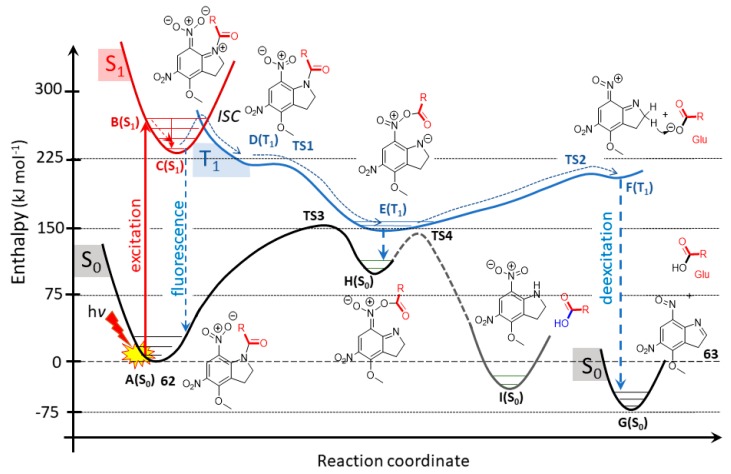
Quantum chemical modelling of the potential enthalpy profile of the singlet (red), triplet (blue) and ground state (black) mechanism for DNI-Glu (**62**) at B3LYP/6-31G(d,p)//PCM(water) level of theory. For detailed data see [138].

The reaction mechanisms of the desired photochemical glutamate release of MNI-Glu (**61**) and DNI-Glu (**62**) were studied and compared on quantum chemical bases using GAUSSIAN 09 software [141] (Figure 24 and Figure 25). Their reaction profile proved to be identical, but the energy values varied in a significant range (4–10 kJ/mol). Here, only the photochemical reaction of DNI-Glu (**62**) will be presented. The photocleavage of DNI-Glu (**62**) starts from the initial state A(S0) and follows the sequence of A → B → C → D → E → F → G. The inadvertent ground state hydrolysis in aqueous media (A → I) also plays an important role during the applications, but will not be discussed here.

After the excitation of the aromatic antenna moiety of DNI-Glu [**62**; A(S_0_)→B(S_1_)] the structure follows a vibrational relaxation [B(S_1_) → C(S_1_)]. The high energy excited singlet state C(S_1_) tends to transform to a triplet state D(T_1_) via an intersystem crossing (ISC) process. The triplet state intermediate takes part in an acyl transfer reaction, where the acyl group migrates from the indoline N atom to one of the O atoms of the nitro group through a low energy barrier (TS1). This process finally results in an unusually stable triplet state [E(T_1_)]. Here, the mechanism may proceed in two different directions. The desired product state, including the free glutamate [Product 1; G(S_0_)], is achieved by the N–O bond cleavage at stage E(T_1_) through TS2, leading to a relatively high energy state F(T_1_). Finally, the deexcitation to ground state [G(S_0_)] results in the final products. However, due to the relative stability of the triplet state E(T_1_), it can undergo a deexcitation process to give a very reactive species H(S_0_), which can return to the starting state A(S_0_).

It is possible to explain the overall process by means of systems chemistry. Through the change in the aromaticity (AR%) [142] and carbonylicity (CA%) values [computed at B3LYP/6-31G(d,p)//PCM(water)] it is possible to understand how the electron distribution follows the energetic changes in the triplet excited as well as the ground state during the photochemical process. DNI-Glu (**62**) is composed of an aromatic ring and a carbonyl group (Figure 24), characterized by values of AR% and CA%. During the excitation [A(S_0_)] → [C(S_1_)] the aromaticity is significantly reduced (AR%: 86.9% → 58.2%), while the carbonylicity value remained relatively constant (CA%: 38.1% → 40.1%). After intersystem crossing (ISC) from singlet to triplet excited state [C(S_1_)]→[D(T_1_)], the molecule tries to regain its stability by slightly increasing its aromaticity (AR%: 58.2% → 66.8%) at the expense of carbonylicity (CA%: 40.1% → 36.1%). The reduced CA% in state D(T_1_) indicates that the amid group is already triggered and favors the next intramolecular rearrangement. This results in a relatively stable triplet state molecule E(T_1_) with an increased aromaticity (77.8%) as well as carbonylicity (41.8%). Subsequently, an endothermic cleavage of the N–O bond leads to stage F(T_1_), then to the product **63** G(S_0_), recovering finally both the AR% and CA% after a de-excitation. During the deexcitation from state E(T_1_) to the ground state G(S_0_), the rearranged molecule loses a lot of stabilization energy, represented by the sharp decreases of AR% (21.8%) and CA% (31.3%), summarized in Figure 24.

The photochemical uncaging process of DNI–Glu is an excellent example of how the electronic excitation of an aromatic moiety can activate a neighboring amide group, leading to an acyl transfer reaction. 

#### 2.5.2. Photochemical Release of Glutamate from Coumarin-Caged Transmitters at Excited State

**Figure 26:**
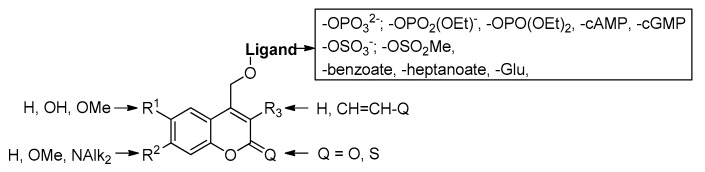
Some examples of cage compounds based on various coumarin scaffolds.

The neurotransmitter derivatives of coumarin-4-yl-methyl have been developed as a new class of efficient caging scaffolds and were applied successfully to mask the biologically active amino, carbonyl [143] and hydroxyl compounds via carbamate [144,145,146] or carbonate [33] linkers. Some phosphate [56,147,148,149,150,151,152,153,154,155], carboxylate [56,155,156], sulfate [154], sulfonate [154] and diol [157] functionalities were also been protected by various coumarin derivatives (Figure 26) [33].

**Figure 27:**
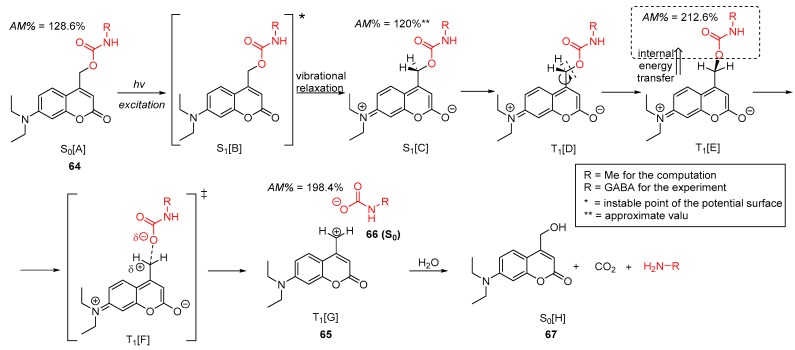
Mechanism of the photochemical initiated scission of coumarin-caged model compound **64** to products **66** + **67** by an internal energy transfer, computed at B3LYP/6-31G(d,p)//PCM(water) level of theory. The amidicity values (AM%) are also shown for some structures.

**Figure 28:**
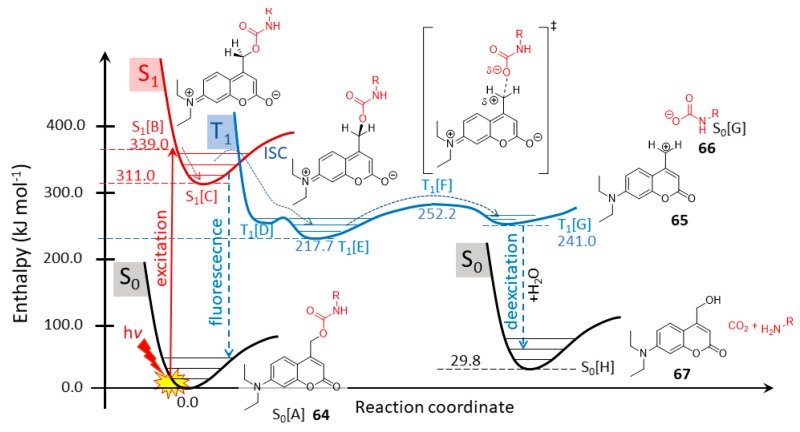
Reaction enthalpy (*H* in kJ mol^−1^) profile of the photochemical initiated scission of coumarin-caged model compound **63** to **65** + **66**, computed at B3LYP/6-31G(d,p)//PCM(water) level of theory.

The photochemical mechanism of diethyl coumarin-Glu cage compound (**64**) has been studied and are reported here by quantum theory (Figure 27) at B3LYP/6-31G(d,p//PCM(water) level of theory. The mechanism and the corresponding enthalpy reaction profile are shown in Figure 28. After the absorption of photon (excitation) by the ground state of coumarin 64 [A(S_0_)], it reaches its excited singlet state B(S1). During the vibrational relaxation process at singlet excited state, the molecule reaches its lowest vibrational energy level C(S1). The molecule tends to deexcite back to its ground state [A(S_0_)] by means of emission (fluorescence) or non-radiative processes. However, some of the excited singlet molecule C(S1) transforms to its triplet state D(T1) via an intersystem crossing (ISC, in Figure 28). In that C(S1) state, the molecule goes through a conformational change, finding the perpendicular arrangement of the side chain carbamate to the aromatic ring as the energy optimum E(T_1_). After a relatively high enthalpy TS [34.5 kJ mol^−1^; F(T_1_)], the CH_2_–O bond is cleaved heterolytically, forming a triplet state coumarin cation (**65**, [G(T_1_)]) and a ground state carbamate anion derivative (66, S_0_). The formation of the real solvent-separated ions is an energy demanding process (estimated as high as +265.4 kJ mol^−1^), so before the ion-pair separation, the positively charged coumarin scaffold deexcites to its ground state and reacts with a water molecule immediately, resulting in coumarin alcohol [66; S_0_(H)]. At the same time, the carbamate derivative **66** yields the desired amino neurotransmitter (in the present cases, Me–NH_2_), while it loses CO_2_. Notably, the overall enthalpy change is endothermic with respect to the starting material in its S_0_ state, however, the reaction is greatly exothermic with respect to the reactant first excited state S_1_[B] or S_1_[C]. 

During the photochemical reaction, the original high amidicity value (AM% = 128.6%) remains nearly constant in its S_1_ state, but in its subsequent triple state (T_1_) its amidicity increased to an unprecedently high value (212.6%), which indicates that the carbamate functionality became a good leaving group, allowing the C–O dissociation. Finally, the forming of ground state carbamate **66** also exhibits a high value. It appears that there has been an internal energy transfer from the excited aromatic ring to the carbamate group. This means that amide, in fact, is stabilized and not activated, as might have been supposed from the study of DNI-Glu in the previous section. 

## 3. Materials and Methods 

All computations were carried out using the Gaussian09 program package (G09) [141]. Geometry optimizations and subsequent frequency analyses were carried out on selected amide-containing systems from which the values for the enthalpy of hydrogenation (Δ*H*_H2_) were extracted. Computations were carried out at B3LYP/6-31G(d,p) level of theory [37]. Method and basis sets were chosen for their reliability in the characterization of amidicity [40] and carbonylicity [20], in agreement with works published earlier. The vibrational frequencies were computed at the same levels of theory as those used for geometry optimization, in order to properly confirm all structures as residing at minima on their potential energy hypersurfaces (PESs). In some cases, the acetonitrile or water solvent was considered by the default IEF-PCM (integral equation formalism polarizable continuum medium) method.

## 4. Conclusions

In general, amide groups have been considered to be very stable and functional for many years, due to their extensive conjugation of the π electron system consisting of the four π electrons. Due to this characteristic, amides can be found frequently in natural or human-made molecules as stable linkages between functioning building blocks, maintaining them for hundreds of years. During synthetic work, they are often and willingly used to inactivate a part of a given molecule as a protecting group. However, as was earlier revealed, the chemistry of the amide bond can be tricked, turning up their reactivity and forming a reactive functional group. 

**Figure 29:**
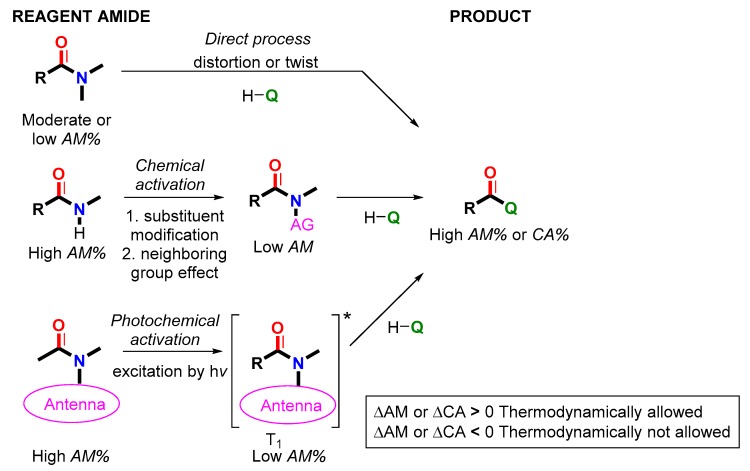
Summary of the amide activation processes. T_1_ = triplet state; AM% = amidicity; CA% = carbonylicity.

In this paper we showed that there is a semi-quantitative rule to predict the outcome of an acyl transfer or transamidation reaction. This thermodynamic selection rule indicates the driving force of amide reactions based on amidicity or carbonylicity values, numerically measuring the amide bond strengths, namely the stabilization enthalpies, toward providing a simple and reliable protocol for practicing chemists. The change of amidicity or carbonylicity in the course of a reaction revealed that the process is favorable or unfavorable (Figure 29). 

The amidicity-based classification of the acyl transfer reactions was demonstrated with a few case examples. Spontaneous processes suppose that the reagent amide exhibits an originally lowered amidicity value, typically by distortion or twisting. If the starting amide exhibits high amidicity it should be lowered by an amide activation. We have shown that it can be achieved by chemical activating groups or photoexcitation, to initiate the acyl exchange. Analogues activation was also used for selective amide reduction. This ON/OFF function of amides can also be revealed in natural biochemical processes, demonstrated by penicillin, blood clotting and intein-mediated protein splicing. 

## Figures and Tables

**Table 1:** The calculated carbonylicity values (CA%) of compounds **17a**–**d** and **18a**–**b** and the carbonylicity changes (ΔCA) for ring-opening alcoholysis depending on the ring size.

State	n = 1 (a)	n = 2 (a)	n = 3 (a)	n = 4 (a)
Start (CA%) 17	46.5%	60.3%	55.2%	53.4%
Product (CA%) 18	56.2%	56.1%	56.1%	56.2%
ΔCA	+10.7%	−4.2%	+0.9%	+2.8%
Method	A or B	only B	A ^1^ or B	A or B

^1^ Extremely slow reaction.
